# Hip dysplasia among children with spastic cerebral palsy in rural Bangladesh

**DOI:** 10.1186/s12891-019-2805-8

**Published:** 2019-10-27

**Authors:** Tasneem Karim, Mahmudul Hassan Al Imam, Prue Golland, Aynul Islam Khan, Iqbal Hossain, Hayley Smithers-Sheedy, Nadia Badawi, Mohammad Muhit, Gulam Khandaker

**Affiliations:** 1 CSF Global, Dhaka, Bangladesh; 2 Asian Institute of Disability and Development (AIDD), University of South Asia, Dhaka, Bangladesh; 3 Discipline of Child and Adolescent Health, Sydney Medical School, The University of Sydney, Sydney, Australia; 4 Cerebral Palsy Alliance Research Institute, The University of Sydney, Sydney, Australia; 5 Singair Upazilla Health Complex, Singair, Manikganj, Bangladesh; 6 Radiology and Imaging Department, Bangabandhu Sheikh Mujib Medical University (BSMMU), Dhaka, Bangladesh; 7 Central Queensland Public Health Unit, Central Queensland Hospital and Health Service, Rockhampton, Queensland Australia

**Keywords:** Hip dysplasia, Cerebral palsy, Children, Bangladesh, Surveillance

## Abstract

**Background:**

Hip dysplasia is common among children with cerebral palsy (CP), particularly in spastic CP. It can result in pain, reduced function and quality of life. However, the burden of hip dysplasia among children with CP in llow-and middle-income countries (LMICs) like Bangladesh is unknown. We aimed to define the burden of hip dysplasia among children with spastic CP in Bangladesh.

**Methods:**

This study includes a subset of the Bangladesh CP Register (BCPR) study cohort who were registered between January and March 2015. The BCPR is a population-based surveillance of children with CP (aged < 18 years) operating in a northern sub-district (Shahjadpur; child population ~ 226,114) of Bangladesh. Community-based key informant’s method (KIM) survey conducted to identify children with CP in the surveillance area. A diagnosis of CP was made based on clinical history and examination by the study physicians and physiotherapist. Study participants had an antero-posterior (AP) X-ray of their pelvis. The degree of subluxation was assessed by calculating the migration percentage (MP).

**Results:**

During the study period, 196 children with CP were registered, 144 had spastic CP. 40 children with spastic CP (80 hips) had pelvic X-Rays (mean age 9.4 years, range 4.0–18.0 years) and 32.5% were female. Gross Motor Function Classification System (GMFCS) showed 37.5% (*n* = 15) with GMFCS level I-II and 62.5% (*n* = 25) with GMFCS level III-V. Twenty percent (*n* = 8) of the children had hip subluxation (MP: 33–80%). Osteopenic changes were found in 42.5% (*n* = 17) children.

**Conclusions:**

To the best of our knowledge this is one of the first studies exploring hip dysplasia among children with spastic CP in Bangladesh. Our findings reflect that hip dysplasia is common among children with spastic CP. Introduction of hip surveillance programmes is imperative for prevention of secondary complications, reduced function and poor quality of life among these children.

## Background

Cerebral palsy (CP) is a heterogenous group of conditions that affects the developing brain, resulting in a permanent non-progressive dysfunction of the central nervous system manifested by disorders of motor function, movement, and posture [1]. It is the leading cause of childhood physical disability globally affecting two to three children per thousand live births [2, 3]. However, the burden of CP is even higher in low-and middle-income countries (LMICs). Recent population-based studies from Bangladesh and Uganda reported population prevalence of CP as 3.4 and 2.9 per 1000 children respectively [4, 5].

The motor disorders of CP are often associated with musculoskeletal anomalies [6], of which hip displacement is the second most common abnormality preceded only by the abnormalities of the foot and ankle. It affects nearly one-third of the children with CP and the incidence varies from 1.0% in children with spastic hemiplegia, up to 75.0% in those with spastic quadriplegia [7, 8]. Progressive hip displacement leading to dislocation can be catastrophic and result in extreme pain, reduced function and quality of life [9, 10]. Other complications include severe contracture, pelvic obliquity and scoliosis, resulting in poor sitting, standing and walking abilities [9, 11, 12]. Most of the studies on hip dysplasia among children with CP are reported from high-income countries, and mostly from areas with an existing hip surveillance programme. However, there is limited data on hip dysplasia from LMICs such as Bangladesh where the burden of CP is substantially high [4].

Studies recommend that radiological screening of children with spastic quadriplegia [13] and GMFCS level IV and V [14] should commence from less than 18 months of age [13]. It is also recommended that children with CP with a migration percentage (MP) of 33 to 40 should undergo radiological examination at 6 months interval prior to surgery [13]. Early detection and management of hip dysplasia can maintain flexible, properly located and pain-free hips with availability of symmetrical range of movement [15]. Introduction of formal surveillance programmes has demonstrated a significant reduction in the incidence of hip dislocation in populations of children with CP in high-income countries [14]. Hip dysplasia and dislocation are preventable through early identification and intervention [8]. However, the burden of hip dysplasia among children with CP in LMICs such as Bangladesh is unknown.

We aimed to describe the burden of hip dysplasia among a population-based cohort of children with CP in rural Bangladesh using the Bangladesh CP Register (BCPR) to inform clinicians and public health professionals [16].

## Methods

### Study design, setting and participants

This study is a part of the BCPR project. BCPR is one of the first population-based CP registers in a LMIC setting and has been ongoing since January 2015 in a northern part of Bangladesh i.e. Shahjadpur (child population ~ 226,114) [17]. Children with CP registered with the BCPR were identified using a novel method, the Key Informant Method (KIM) which involves training of local volunteers to serve as key informants (KIs) and list children with CP from their community. The children then undergo detailed clinical assessment by a multidisciplinary team for confirmation of diagnosis and registration into the BCPR. The details of the BCPR study has been described previously [4, 16].

Between January and March 2015, a sub-group of the BCPR registrants were included in this study. Due to limited available resources, every third child from the BCPR cohort with spastic CP was selected for radiological assessment. In the event that any prospective study participant was unwilling the next child was approached for participation in the study.

### Clinical assessment

Children with CP were identified from the community using the KIM, [18] and a detailed neurological examination was performed by a trained paediatrician and physiotherapist during the clinical assessment and BCPR registration process. Sociodemographic and clinical data were collected using BCPR record forms. Motor type and motor severity including Gross Motor Functional Classification System (GMFCS) levels were determined following standard guideline [19]. The predominant motor type of CP included spastic, dyskinetic or ataxic movement motor types based on clinical assessment by a paediatrician. Spastic topography was further classified as mono/hemiplegic, diplegic, triplegic, and quadriplegic.

### Radiological assessment

Children with spastic CP had an anteroposterior (AP) pelvic X-ray. Hip displacement was measured from an AP radiograph of the hips using existing protocol [20]. The degree of subluxation (dysplasia) was assessed by calculating the MP i.e. the percentage of the bony width of the femoral capital epiphysis which falls lateral to a line drawn vertically from the bony lateral margin of the acetabulum, the Perkins line. This technique has been found useful to determine hip dysplasia among children [21]. Studies of validity have shown that a change greater than 8.3% in MP represents a real change in displacement of the femoral head with 95% confidence [20]. The MP found among the subjects were classified as normal hip (MP: <32%), and subluxated hip (MP: 33 – 80%) [22]. Radiological diagnosis of osteopenia was made by the radiologist after qualitative assessment of the X rays. Qualitative features of osteopenic bone included increased radiolucency, vertical striation, end plate thinning and accentuation of cortical margins of any vertebra including changes in shape.

### Ethical consideration

The study was approved in Australia by the Cerebral Palsy Alliance NHMRC Human Research Ethics Committee (Ref no. 2015–03–02), and in Bangladesh by the Asian Institute of Disability and Development (AIDD) Human Research Ethics Committee (southasia-irb-2014-l-01) and Bangladesh Medical Research Council (BMRC) HREC (BMRC/NREC/2013–2016/1267). Informed written consent was obtained from the primary caregiver of the children prior to recruitment into the study.

### Statistical analysis

The data were processed using SPSS (Statistical Package for Social Science, version 22.0 for windows; SPSS Inc., Chicago, IL, USA). The measures of central tendency (mean) and dispersion (SD) were used. Descriptive analyses were done to present the proportion of dysplastic hips according to the background factors (age, sex, parental education), and degree of motor impairment of the children with CP.

## Results

Out of the 196 BCPR registrants during the study period, 73.4% (*n* = 144) had spastic CP of which 40 children underwent radiological assessment. The mean age was 9.4 ± 4.0 years (range 4.0–18.0 years) and 32.5% (*n* = 13) of the study cohort were female.

The majority of the mothers had completed primary education (42.5%, *n* = 17) whereas more than one third of the fathers had not have any education (37.5%, *n* = 15). The median age of diagnosis of CP was 3 years. The majority (37.5%, *n* = 15) of the children had diplegia. 37.5% (*n* = 15) were described as GMFCS level I-II and 62.5% (*n* = 25) as GMFCS level III-V. Large numbers of children with CP (60%, *n* = 24) had not ever received rehabilitation services. Twenty percent (*n* = 8) of the children had hip subluxation of which five children were between 5 to 9 years of age (Table 1).

**Table 1:** Socio-demographic characteristics, age of CP diagnosis, neurological subtype of Spastic CP, gross motor function classifications and rehabilitation status of children with CP

Characteristics	*n* (%)
Gender	
Male	27 (67.5)
Female	13 (32.5)
Age (years)	
< 5	6 (15.0)
5–9	18 (45.0)
10–14	12 (30.0)
15–18	4 (10.0)
Maternal education	
Illiterate	11 (27.5)
Primary	17 (42.5)
Secondary	9 (22.5)
More than Secondary	3 (7.5)
Paternal education	
Illiterate	15 (37.5)
Primary	10 (25.0)
Secondary	10 (25.0)
More than Secondary	5 (12.5)
Age of diagnosis of CP (months)	
0–23	1 (2.5)
24–47	21 (52.5)
48–84	11 (27.5)
85 & above	7 (17.5)
Spastic subtype	
Hemi/monoplegia	11 (27.5)
Diplegia	15 (37.5)
Triplegia	4 (10.0)
Quadriplegia	10 (25.0)
GMFCS levels	
Level I	5 (12.5)
Level II	10 (25.0)
Level III	12 (30.0)
Level IV	4 (10.0)
Level V	9 (22.5)
Received rehabilitation services	
Yes	16 (40.0)
No	24 (60.0)

### Hip subluxation and age CP diagnosis, type of CP, GMFCS and rehabilitation status

The majority (75.0%, *n* = 6) of the children who had subluxation of hips were diagnosed with CP between 24 to 47 months of age. Children with subluxated hips had spastic diplegia (50.0%, *n* = 4), quadriplegia (37.5%, *n* = 3) and hemiplegia/monoplegia (12.5%, *n* = 1). Children with GMFCS levels III -V had the highest proportion of subluxated hips; 37.5% (n = 3). Among the children with hip subluxation 62.5% (*n* = 5) never accessed any rehabilitation services (Table 2).

**Table 2:** Distribution of hip displacement according to socio-demographic factors, spastic CP sub-type, GMFCS and rehabilitation status

	Right		Left		Total	
Age	Normal	Subluxated	Normal	Subluxated	Normal	Subluxated
0–4 years (*n* = 6)	5 (14.3)	1 (20.0)	5 (13.5)	1 (33.3)	10 (13.9)	2 (25.0)
5–9 years (*n* = 18)	14 (40.0)	4 (80.0)	17 (45.9)	1 (33.3)	31 (43.1)	5 (62.5)
10–14 years (*n* = 12)	12 (34.3)	0 (0.0)	11 (29.7)	1 (33.3)	23 (31.9)	1 (12.5)
15 and above (n = 4)	4 (11.4)	0 (0.0)	4 (10.8)	0 (0.0)	8 (11.1)	0 (0.0)
Maternal Education						
Illiterate (*n* = 11)	11 (31.4)	0 (0.0)	11 (29.7)	0 (0.0)	22 (30.6)	0 (0.0)
Primary (*n* = 16)	15 (42.9)	2 (40.0)	15 (40.5)	2 (66.7)	30 (41.7)	4 (50.0)
Secondary (*n* = 9)	7 (20.0)	2 (40.0)	8 (21.6)	1 (33.3)	15 (20.8)	3 (37.5)
More than Secondary (*n* = 3)	2 (5.7)	1 (20.0)	3 (8.1)	0 (0.0)	5 (6.9)	1 (12.5)
Paternal Education						
Illiterate (*n* = 15)	14 (40.0)	1 (20.0)	15 (40.5)	0 (0.0)	29 (40.3)	1 (12.5)
Primary (*n* = 10)	9 (25.7)	1 (20.0)	9 (24.3)	1 (33.3)	18 (25.0)	2 (25.0)
Secondary (*n* = 10)	7 (20.0)	3 (33.3)	9 (24.3)	1 (33.3)	16 (16.7)	4 (50.0)
More than Secondary (*n* = 5)	5 (14.3)	0 (0.0)	4 (10.8)	1 (33.3)	9 (12.5)	1 (12.5)
Age of diagnosis of CP (months)						
0–23 (*n* = 1)	1 (2.9)	0 (0.0)	1 (2.7)	0 (0.0)	2 (2.8)	0 (0.0)
24–47 (*n* = 21)	17 (48.6)	4 (80.0)	19 (51.4)	2 (66.7)	36 (50.0)	6 (75.0)
48–84 (*n* = 10)	10 (28.6)	1 (20.0)	10 (27.0)	1 (33.3)	20 (27.8)	2 (25.0)
85 & above (*n* = 7)	7 (20.0)	0 (0.0)	7 (18.9)	0 (0.0)	14 (19.4)	0 (0.0)
Spastic subtype						
Hemiplegia/ monoplegia (*n* = 11)	11 (31.4)	0 (0.0)	10 (27.0)	1 (33.3)	21 (29.2)	1 (12.5)
Diplegia (*n* = 14)	12 (34.3)	3 (60.0)	14 (37.8)	1 (33.3)	26 (36.1)	4 (50.0)
Triplegia (*n* = 4)	4 (11.4)	0 (0.0)	4 (10.8)	0 (0.0)	8 (11.1)	0 (0.0)
Quadriplegia (*n* = 10)	8 (22.9)	2 (40.0)	9 (24.3)	1 (33.3)	17 (23.6)	3 (37.5)
GMFCS						
I-III (*n* = 27)	25 (71.5)	2 (40.0)	25 (67.5)	2 (66.6)	50 (69.4)	4 (50.0)
IV-V (*n* = 13)	10 (28.6)	3 (60.0)	12 (32.4)	1 (33.3)	22 (30.5)	4 (50.0)
Received rehabilitation services						
Yes (*n* = 24)	23 (65.7)	1 (20.0)	22 (59.5)	2 (66.7)	45 (62.5)	3 (37.5)
No (*n* = 16)	12 (34.3)	4 (80.0)	15 (40.5)	1 (33.3)	2 (37.5)	5 (62.5)

### Qualitative findings

On qualitative assessment of the x-rays by a radiologist we found that 42.5% (*n* = 17) of the children had signs of osteopenic changes of which 94.1% (*n* = 16) had mild osteopenia and 5.8% (*n* = 1) had moderate osteopenic changes.

## Discussion

The aim of the study was to investigate the burden of hip dysplasia among children with spastic CP from a population-based CP register in rural Bangladesh. We found that a fifth of our cohort had subluxation of hips which has yielded valuable insights into the high burden of hip dysplasia among children with spastic CP in Bangladesh.

Subluxation of hips was more commonly observed among children over 5 years of age and with more severe functional motor limitations (i.e. GMFCS levels III - V). This is consistent with other studies from high income countries which showed that the more severe outcomes of hip dysplasia are observed among older children and among those with higher GMFCS levels [7, 14, 23]. Furthermore, all the children with subluxation of hips in our cohort were diagnosed with CP after 24 months of age and nearly two thirds of them had not ever received any rehabilitation services.

Findings from our study have a bearing on the importance of hip surveillance among children with CP in LMIC settings. Hip dysplasia can be detected early by monitoring through regular hip X-rays to facilitate timely treatment and can thereby avert complications which are detrimental to the quality of life of children with CP and their families [9, 10]. In contrast to high income countries, where a range of treatment options are available for the management of of children with CP, including botulinum toxin injection, bracing, soft tissue release surgery for spasticity, reconstructive surgeries and salvage procedure [24], the prognosis of children with CP is remarkably different in poorer countries where there is limited access to such services. In addition to the lack of services these children and their families face additional challenges due to socioeconomic factors and rampant stigma around disabilities such as CP [25].

The position of femoral head within the acetabulum up to the age of 5 years is important to ensure stability of the hip and acetabular development [26, 27]. Therefore, early detection of progressively displaced hips and referral for orthopedic management is imperative to prevent further displacement and to keep the femoral head in position within the age of 5 years [28]. However, early diagnosis of hip dysplasia is challenging particularly through physical examination alone. Regular clinical inspection for the range of abduction of the hip, and repeated radiological examination of the hips is necessary to identify hip dysplasia in time [8, 29, 30]. The aim of hip surveillance programmes that are based on the Consensus Statement on Hip Surveillance for Children with Cerebral Palsy is to ensure that progressive hip displacement is detected early enough to enable timely referral for orthopedic assessment and management [31]. Decrease in incidence of hip dislocation has been previously documented with the introduction of hip surveillance programme [13]. Incorporation of hip surveillance into routine care has been recommended for all children with CP. This includes radiological assessment between 12 to 24 months of age with subsequent management varying with gross motor severity i.e. GMFCS levels [14]. Studies also suggested that non-ambulatory children and those who have an annual increment of the MP by more than 7% require more vigilant monitoring and consideration for orthopedic referral [1, 32].

A study conducted in Norway and Southern Sweden compared the burden of hip dislocation among children with CP in regions with and without hip surveillance programmes [20]. The sample size at the two sites (*n* = 119 in Norway and *n* = 136 in Southern Sweden) were comparable to our study cohort of 196 children with CP. The proportion of children at GMFCS levels III-V among both the study populations were substantially lower than our cohort; 34.0% in Norway and 38.0% in Southern Sweden population compared to 62.5% in our study cohort in Bangladesh [20]. It was observed that in Southern Sweden where hip surveillance services are provided the prevalence of hip dislocation was lower, fewer children required surgical intervention and the children underwent hip operations at an earlier age [20]. 20.0% of the children from our cohort had hip subluxation which is comparable to the findings from Norway (15.1%). Whereas Southern Sweden had a significantly lower proportion (0.7%, *p* < 0.001) of children with hip subluxation in presence of a surveillance programme [20].

Early identification of hip dysplasia is limited by the absence of hip surveillance programmes, thereby resulting in a high burden [31, 32]. A recent study conducted in India among 118 children with CP aged 2 to 12 years with GMFCS levels III-V showed that 12.7% had developmental dysplasia of spastic hip [21]. An even higher burden of hip dysplasia (54.7%) was reported in another study conducted in Malaysia [33].

Findings from these studies collectively illustrate the apparent benefits of hip surveillance programmes in the reduction of the burden of hip dysplasia and the aversion of associated complications which drastically impair the wellbeing of children with CP [20, 21, 33]. There is a dire need for prospective hip surveillance programmes for children with CP in LMICs. Our study has direct clinical implication; the established programmes in high income countries and the method described in this study can be incorporated for the management of children with CP in LMICs. As radiological services are becoming increasingly available in semi-rural areas of LMICs such as Bangladesh, it is now possible to develop and implement hip surveillance programmes with a simplified protocol for these settings. Ongoing surveillance of children with CP such as the BCPR can serve as the basis for the implementation of recommended guidelines for hip dysplasia among children with CP in low resource settings. This will ensure early detection of hip dysplasia and subluxation which can thereby facilitate early intervention to avert and limit the adverse outcomes of hip dysplasia.

### Study limitations

Despite our best efforts there are several limitations in this study which are inherent to observational studies. The cross-sectional design limited the establishment of causal relations between the independent variables and hip dysplasia. Radiography of hips were undertaken in a position as neutral as possible which was often difficult to achieve owing to spasm or contractures. We had to restrict or study only among children with spastic CP due to budget constraints. However, studies have shown that children with spastic CP are the most affected by hip dysplasia. Advanced statistical analyses were also limited due to the small number of children in subgroups (i.e. eight children with subluxation). Furthermore, caution is recommended in the interpretation of comparisons between studies due to methodological differences.

## Conclusion

To the best of our knowledge this is one of the first studies exploring hip dysplasia among children with spastic CP in Bangladesh. Our findings from this selected sample suggest hip subluxation is common among children with spastic CP in rural Bangladesh. Further studies are needed to address the true extent of this complex complication of CP to improve the quality of life and ensure better prognosis among children with CP and their families. Introduction of hip surveillance programmes is imperative for the prevention of secondary complications, reduced function and poor quality of life among these children.

## Data Availability

The datasets generated and/or analysed during the current study are not publicly available due to confidentiality but are available from the corresponding author on reasonable request.

## Abbreviations

AP: Antero-posterior
BCPR: Bangladesh CP Register
CP: Cerebral Palsy
GMFCS: Gross Motor Function Classification System
KIM: Key Informant Method
KIs: Key Informants
LMICs: Low and Middle-Income Countries
MP: Migration Percentage
